# Proteomics analysis of metabolically engineered yeast cells and medium-chained hydrocarbon biofuel precursors synthesis

**DOI:** 10.1186/s13568-014-0061-8

**Published:** 2014-08-21

**Authors:** Xiang Li, Wei Ning Chen

**Affiliations:** School of Chemical and Biomedical Engineering, Nanyang Technological University, 62 Nanyang Drive, Singapore, 637459 Singapore

**Keywords:** Saccharomyces cerevisiae, Lipoxygenase, Hydroperoxide lyase, Medium-chained biofuel precursors, Proteomics

## Abstract

Recently, various biofuels have been synthesized through metabolic engineering approaches to meet the exploding energy demands. Hydrocarbon biofuels, energy-equivalent to petroleum-based fuels, are identified as promising replacements for petroleum. Metabolically engineered *Saccharomyces cerevisiae* capable of synthesize precursors of medium-chained hydrocarbons is proposed in this study. The hydroperoxide pathway introduced in *S. cerevisiae* consisted of lipoxygenase (LOX) and hydroperoxide lyase (HPL) from almond, which catalyzes linoleic acid to 3(*Z*)-nonenal, the precursor for medium-chained hydrocarbon biofuels. Proteomics study showed that 31 proteins displayed different expression levels among four functional strains and most of them were related to carbohydrate metabolism and protein synthesis, suggested prospective capabilities of energy generation and exogenous protein synthesis. Biotransformation efficiency studies carried out by GC-FID were in accordance with the expectations. The highest yield of 3(*Z*)-nonenal was up to 1.21 ± 0.05 mg/L with the carbon recovery of up to 12.4%.

**Electronic supplementary material:**

The online version of this article (doi:10.1186/s13568-014-0061-8) contains supplementary material, which is available to authorized users.

## Introduction

Recently, petroleum shortage and environmental concerns have emphasized the synthesis and utilization of renewable fuels (Atsumi et al. [2008]; Chang and Keasling [2006]; Lennen et al. [2010]). Biomass-derived ethanol as drop-in fuel is currently in use, while hydrocarbons which eliminate the drawbacks of ethanol are also promising biofuels (Regalbuto [2009]).

Hydrocarbons are highly compatible with existing energy infrastructure due to its chemical resemblance to traditional petroleum-based fuels. Besides, hydrocarbons are energy-equivalent to petroleum-based fuels and render no mileage penalty in the procedure of usage. Moreover, being immiscible in water eliminate the additional effort required for water separation and distillation step (Boundy et al. [2011]), further makes hydrocarbons promising diesel substitutes.

Recent research has identified various genetically-engineered micro-organisms capable of producing hydrocarbons (Steen et al. [2010]; Rutherford et al. [2010]). Fatty aldehydes derived from lipid biosynthesis were identified to be metabolically flexible precursors for a diversity of biofuels, including alkanes, free fatty acids and wax esters (Kaiser et al. [2013]). In this study, we will therefore explore the biosynthesis capabilities of medium-chained aldehydes through metabolic engineering approaches.

**Figure 1:**
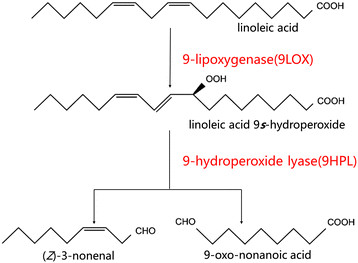
**Pathway of medium-chained biofuels precursor synthesis.**

The aldehyde-producing hydroperoxide pathway in plants has been studied and the corresponding genetic information has been elucidated (Mita et al. [2001]; Santino et al. [2005]; Tijet et al. [2001]; Mita et al. [2005]). Hydroperoxide pathway starts with hydroperoxidation of polyunsaturated fatty acid, linoleic acid. With LOX catalyzing, one peroxy is inserted onto the backbone of linoleic acid and yield one unsaturated acid hydroperoxide (HPOD). HPOD can subsequently be metabolized via a number of secondary reactions while one of them is to be cleaved by HPL and yield one aldehyde and one oxo-acid (Feussner and Wasternack [2002]). Linoleic acid can be oxygenated either at carbon atom 9(9LOX) or 13(LOX) of the backbone. In the case of oxygenating at carbon atom position 9, linoleic acid will be diverted to 3(*Z*)-nonenal and 9-oxo-nonanoic acid, as shown in Figure 1. 3(*Z*)-nonenal is our target medium-chained biofuel precursor in this study. We used *S. cerevisiae* to construct whole-cell based catalyst which was capable of synthesize 3(*Z*)-nonenal through exogenous expressing of 9LOX and 9HPL from almond (*Prunus dulcis*).

It has been reported that, after absorption into *S. cerevisiae* from media, the degradations of long-chained fatty acids (LCFAs) are confined in peroxisomes (Hiltunen et al. [2003]; Hettema and Tabak [2000]). The protein complex, Pxa1p-Pxa2p, which embeds in the peroxisomal membrane, functions as transporter and translocates activated fatty acids into peroxisomes for beta-oxidation, utilizing the energy of ATP hydrolysis. Furthermore, previous results have confirmed that LCFAs cannot enter peroxisomes in Δ*pxa1* and Δ*pxa2* mutant and disruption of either pxa1 or pxa2 leads to latency of LCFA β-oxidation while disrupting both genes exhibited similar phenotype (Hettema et al. [1996]).

In this study, exogenous genes 9LOX and 9HPL were expressed in *S. cerevisiae*. Apart from wild type as control, we used Δ*pxa1*, Δ*pxa2* and Δ*pxa1&2* mutants as the hosts to block the translocations of absorbed LCFAs into peroxisomes and divert them to the exogenous hydroperoxide pathway. Proteomics analysis using 2D LC-MS/MS approaches provided us a global overview of protein expression levels to determine the potentials of the constructed whole-cell based catalysts (Wiese [2007]). The biotransformation efficiencies of the functional strains were also characterized by GC-FID approach and the highest yield of 3(*Z*)-nonenal we achieved was up to 1.21 mg/L.

## Materials and methods

### Strains and culture media

*E.coli* strain Top10 was used for cloning and plasmid propagation and cultured at 37°C with constant shaking at 250 rpm. LB broth contained 10 g/L bacto-tryptone (Fluka), 5 g/L yeast extract and 5 g/L NaCl (Sigma). The *S. cerevisiae* strains (Table 1) were cultured at 30°C with constant shaking at 250 rpm. YPD medium consisted of 10 g/L yeast extract (Fluka), 20 g/L peptone (Bacto) and 20 g/L dextrose (Sigma). YNB-LEU selective media consisted of 6.7 g/L yeast nitrogen base without amino acids (Sigma), 0.69 g/L DO Supplement-LEU (Clontech) and 20/L dextrose or galactose (Sigma). YNB-HIS selective media contained 6.7 g/L yeast nitrogen base without amino acids (Sigma), 0.69 g/L DO Supplement-HIS (Clontech) and 20/L dextrose or galactose (Sigma).

**Table 1:** **Primers, plasmids and strains used in this work**

Name	Description	Reference
***E. coli*** **strain**		
Top 10	F-*mcr* A Δ(*mrr*-*hsd* RMS-*mcr* BC) Φ80*lac* ZΔM15 Δ*lac* X74 *rec* A1 *ara* D139 Δ(*ara leu*) 7697 *gal* U *gal* K*rps* L (StrR) *end* A1 *nup* G	Life Technology
**Primers for gene disruption**		
Δ*pxa2*-F	5′- ATAATAATAC AATTAAAAGT TACCGAAGAA AGATTTTATA CAGCTGAAGC TTCGTACGC-3′	This work
Δ*pxa2*-R	5′- CAATTTATAC ATGATTTGGA TCCTCCTTTG GCTATGTATG GCATAGGCCA CTAGTGGATC TG-3′	This work
**Primers for restriction endonuclease**		
F-*BamHI*	5′-CG*GGATCC* AT GTTGCATAAC TTGTTCGACA AGA-3′	This work
R-*SalI*	5′-GC*GTCGAC* AG TAGAATCCAA ACCCAACAAT GGA-3′	This work
**Plasmids**		
PUG27	*loxP*-*kanMX*-*loxP* disruption module plasmid	EUROSCARF
pESC-*leu*	With *GAL1* and *GAL10* yeast promoters in opposite orientation, CYC1 and ADH1 terminator respectively	Agilent
***S. cerevisiae*** **strains**		
Wild type	*MATa; his3Δ 1; leu2Δ 0; met15Δ 0; ura3Δ 0*	EUROSCARF
Δ*pxa1*	*BY4741; Mat a; his3D1; leu2D0; met15D0; ura3D0; YPL147w::kanMX4*	EUROSCARF
Δ*pxa2*	*BY4741; Mat a; his3D1; leu2D0; met15D0; ura3D0; YKL188c::kanMX4*	EUROSCARF
Δ*pxa1&2*	*BY4741; Mat a; his3D1; leu2D0; met15D0; ura3D0; YPL147w::kanMX4; YKL188c::his*	This work
WT-pESC	wild type carring pESC	This work
Δ*pxa1*-pESC	Δ*pxa1* carrying pESC	This work
Δ*pxa2*-pESC	Δ*pxa2* carrying pESC	This work
Δ*pxa1&2*-pESC	Δ*pxa1&2* carrying pESC	This work
WT-9LHP	wild type carring 9LHP	This work
Δ*pxa1*-9LHP	Δ*pxa1* carrying 9LHP	This work
Δ*pxa2*-9LHP	Δ*pxa2* carrying 9LHP	This work
Δ*pxa1&2*-9LHP	Δ*pxa1&2* carrying 9LHP	This work

### Recombinant plasmid construction

All the oligonucleotide primers in Table 1 were synthesized by Integrated DNA Technologies. All restriction enzymes used in this study were purchased from New England Biolabs. Ligation reactions were performed using T4 ligase (Fermentas). PCR reactions were carried out with HotStarTaq *Plus* Master Mix Kit (Qiagen) according to standard protocols. Gel extractions were carried out using QIAquick Gel Extraction Kit (Qiagen). *E.coli* minipreps were performed with QIAprep Spin Miniprep Kit (Qiagen).

Codon optimized genes 9LOX and 9HPL were generated by Geneart (acc. No. KC920894 and KC920895). The cloning vector pESC-LEU (Agilent) was adopted, which contains the *GAL1* and *GAL10* yeast promoters in opposing orientations, capable of introducing two genes into one strain under the control of a repressible promoter.

Primers F-*BamHI* and R-*SalI* in Table 1 were used to introduce *BamHI* and *SalI* into 9LOX. Flanked by 5′ *BamHI* restriction enzyme site and 3′ *SalI* site, 9LOX gene was then inserted into pESC plasmid to obtain 9LOX-pESC recombinant plasmid. *SacI* and *NotI* restriction endonucleases were adopted to double digest 9HPL gene from default plasmid pMK-RQ. The DNA fragment is flanked by 5′ *SacI* restriction enzyme site and 3′ *NotI* site, and inserted into 9LOX-pESC recombinant plasmid to obtain the recombinant plasmid 9LOX-9HPL-pESC (9LHP), shown in Additional file 1: Figure AF2.

### Double deletion strain construction

The pUG plasmid carrying gene disruption cassettes containing HIS5 heterologous marker genes with *loxP* sites was selected for gene disruption (Gueldener et al. [2002]). The target genes in *S. cerevisiae* were pxa1 and pxa2, the heterodimers of peroxisomal membrane transporter Pxa1p-Pxa2p. The sequences flanking the target genes were added to the 5′ end of OL3′ and OL3′ sequences: 40 nucleotide stretches that are homologous to sequences upstream of the ATG start codon, and down-stream of the stop codon of the targeted gene respectively. Primer sequences are shown in Table 1.

*S. cerevisiae* wild type, Δ*pxa1* and Δ*pxa2* strains were purchased from EUROSCARF. The Δ*pxa1* strain was transformed with the *pxa2::his* using PEG-LiAc method (Gietz and Schiestl [2007]) to construct double deletion strain Δ*pxa1&2*.Transformed deletion stains Δ*pxa1&2* strain were selected via histidin prototroph by growing on synthetic complete minimal medium deficient in histidine. Yeast colony PCR was carried out to further confirm the gene disruption.

### Functional strains construction

Recombinant plasmid 9LHP was transformed into *S. cerevisiae* wild type, Δ*pxa1*, Δ*pxa2* and Δ*pxa1&2*, to obtain WT-9LHP, Δ*pxa1*-9LHP, Δ*pxa2*-9LHP and Δ*pxa1&2*-9LHP functional strains. Corresponding controls, WT-pESC, Δ*pxa1*-pESC, Δ*pxa2*-pESC and Δ*pxa1&2*-pESC were constructed by transforming empty pESC plasmid into the four strains (Table 1).

### Protein extraction and labeling

The *S. cerevisiae* functional strains WT-9LHP, Δ*pxa1*-9LHP, Δ*pxa2*-9LHP and Δ*pxa1&2*-9LHP were cultured at 30°C with constant shaking at 250 rpm using 50mLYPD-LEU selective media containing galactose to induce the promoter. After 3 days’ culture, *S. cerevisiae* cells were collected. For cell lysis and protein extraction, all steps were carried out on ice to avoid denaturation of proteins. Same amount (OD_600_ = 20) units of yeast cells were pelleted at 13,000 rpm, 4°C for 5 min. The cell pellets were washed twice by distilled water and re-suspended in 300 μL of yeast lysis buffer which consisted of: 8 M Urea, 50 mM DTT, 50 mM Tris-Cl (pH7.6), 100 mM NaCl, 0.1% Triton X-100, 1 mM EDTA and 1 mM PMSF. Equal volumes of acid-washed glass beads were added and the mixtures were performed in the bead mill by 5 cycles of 30s of vortex at 4.0 m/s with 30s of cooling on ice. Lysates were centrifuged at 10,000 rpm for 10 min at 4°C and supernatants were collected and stored at −80°C. The protein concentrations were determined following the standard protocol of 2D Quant Kit (GE Healthcare).

A total of 100 μg proteins from functional strains WT-9LHP, Δ*pxa1*-9LHP, Δ*pxa2*-9LHP and Δ*pxa1&2*-9LHP were collected and labeled by iTRAQ Reagent Multi-Plex Kit (AB Sciex) according to the standard protocol as follows: 20 μL dissolution buffer and 1 μL denaturant were added to each sample; vortex to mix; 2 μL reducing reagent was added to each sample; incubation at 60°C for 1 h; 1 μL cysteine-blocking reagent was added to each sample; vortex to mix; incubate 10 min at room temperature; 20 μL of 0.25 μg/μL sequence grade modified trypsin (Promega, US) was added to each sample to digest the protein overnight at 37°C; amino-modifying labeling reagent 114, 115, 116 and 117 were used to label four samples respectively: WT-9LHP protein sample was labeled with iTRAQ tag 114; Δ*pxa1*-9LHP protein protein sample was labeled with iTRAQ tag 115; Δ*pxa2*-9LHP protein sample was labeled with iTRAQ tag 116; Δ*pxa1&2*-9LHP protein sample was labeled with iTRAQ tag 117. The labeled samples were then combined together and condensed to roughly 100 μL using a thermal shaker at 30°C.

### LC-MS analysis

The labeled samples were analyzed by online 2D Nano-LC-MS/MS 1200 series nanoflow liquid chromatography system (Agilent Technologies) interfaced with 6500 Q-TOF mass-spectrometer with HPLC-Chip Cube (Agilent Technologies). The HPLC-Chip was a combination of Zorbax 300SB C_18_ reversed-phase column (75 μm × 50 mm, 3.5 μm) packing with Zorbax 300SB C_18_ enrichment column (0.3 × 5 mm, 5 μm).

In the first dimension, 4 μL of sample was loaded onto the polysulfoethyl, a strong cation-exchange (SCX) column (0.32 × 50 mm, 5 μm). The retained peptides were then eluted by injecting 8 μL ammonium formate solutions in concentration gradient of 20, 40, 60, 80, 100, 200, 500 and 1000 mM. In the second dimension, the effluent was trapped onto Zorbax 300SB C_18_ enrichment column during the enrichment mode by buffer A (5% acetonitrile and 0.1% formic acid) with a flow rate of 4 μL/min. Then the peptides trapped on enrichment column were eluted for 60 min by buffer B (0.1% formic acid) and buffer C (0.1% formic acid + acetonitrile nanoflow gradient from 5% to 80% in 60 min) at a flow rate of 300 μL/min. Subsequently, the effluent flowed through the analytical Zorbax 300SB C_18_ reversed-phase column for separation with the HPLC-Chip on analytical mode. The analysis was accomplished by 6500 Q-TOF mass spectrometer with a capillary voltage of 1950 V for 10 runs in total. For MS analysis, positive ionization mode was used. Survey scans were from m/z 300 to 2000 with an acquisition rate of 4 spectra per second.

### LC-MS/MS data analysis

Peptide quantification and protein identification were performed with Spectrum Mill MS Proteomics Workbench (Agilent Technologies). Each MS/MS spectrum was searched for the species of *S. cerevisiae* against the UniProt-Swiss-Prot database. Methyl-methane-thiosulfate-labeled cysteine and iTRAQ modification of free amine in the amino terminus and lysine were set as fixed modification. Protein relative quantification using iTRAQ was performed on the MS/MS scans. Protein quantification data with two or more unique peptides identified with confidence > 99% and the p value < 0.05 were selective for further statistical analysis. Three independent batches were performed to increase statistically evidence of protein expression. The overlapping isotopic contributions were used to correct the calculated peak area ratios and to estimate the relative abundances of a specific peptide.

### Biotransformation product detection

Functional strains were cultured in YNB-LEU containing galactose to induce the promoters. After reaching an OD_600_ = 1, 20 mL of the culture was collected and pelleted and washed twice with 100 mM potassium phosphate buffer at pH = 6.5 to prepare resting cells and then transferred to 250 mL GL-45 Erlenmeyer flask (Chemglass Life Sciences). Biotransformation buffer used was 20 mL potassium phosphate buffer with 100 μL linoleic acid solution (5% v/v with 0.2% tween-80). The flasks were sealed with GL-45 open top cap and parafilms (Chemglass Life Sciences) and incubated at 30°C on an orbital shaker (250 rpm) for 3 days.

Headspace samples of the cultures were determined by Agilent 6890 N GC-FID system (Agilent) equipped with Agilent J&W DB-WAX column (30 m × 0.25 mm × 0.25 μm, Agilent). 1 mL SampleLock syringe (Hamilton) was used to draw out the headspaces of the 20 mL cultures to inject into GC system. GC settings were: carrier gas: helium; column flow: 2.0 ml/min; splitless; inject temperature: 230°C The analyzing temperature program used was: 50-230°C in 18 min; 230°C for 2 min. Product identification was carried out by comparing with authentic standards and benzoaldehyde was used as internal control for quantification.

## Results

### Strains construction

Recombinant plasmid 9LHP (shown in Additional file 1: Figure AF2) was constructed according to the procedure described in “Materials and methods”. The size of the recombinant plasmid 9LHP was 11820 bp and gene-sequencing results proved that no site mutation in the recombinant plasmid.

We constructed double mutant Δ*pxa1&2* using PUG27 plasmid carrying *lox-his5*^*+*^*-lox*. Then recombinant plasmid 9LHP or empty vector pESC was transformed into *S. cerevisiae* strains wild type, single mutants Δ*pxa1*-9LHP and Δ*pxa2*-9LHP and double mutant Δ*pxa1&2* to obtain functional strains WT-9LHP, Δ*pxa1*-9LHP, Δ*pxa2*-9LHP, Δ*pxa1&2*-9LHP and control strains WT-pESC, Δ*pxa1*-pESC, Δ*pxa2*-pESC, Δ*pxa1&2*-pESC. All the functional strains and control strains grown well on YPD and YNB-LEU selective minimal media.

### Proteomic analysis

**Figure 2:**
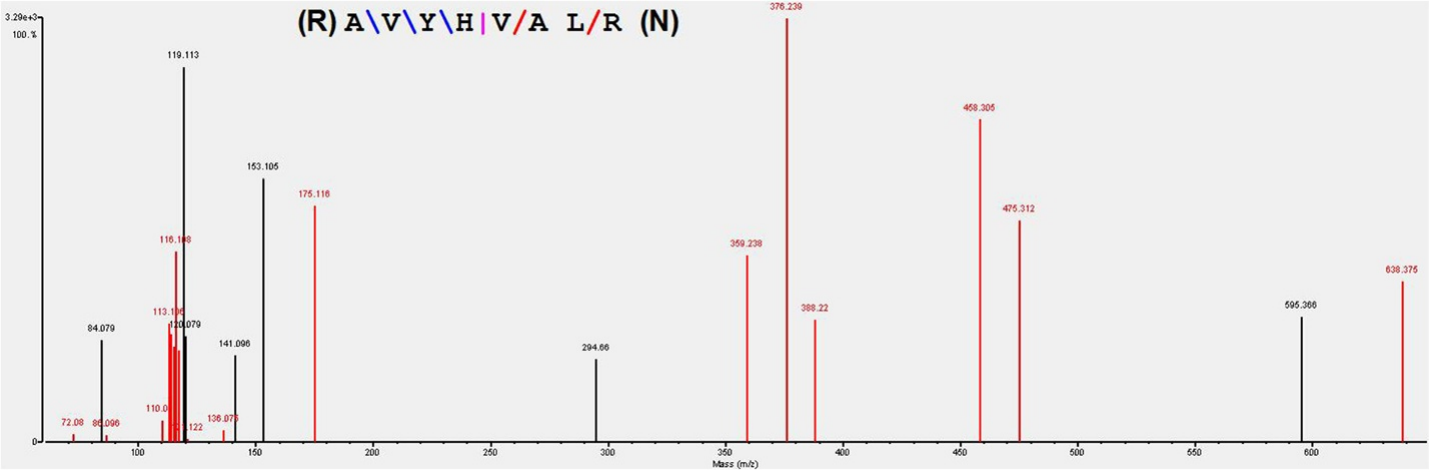
**Representative peptide fragmentation spectrum of glucose-6-phosphate isomerase: (R)AVYHVALR(N) in WT-9LHP,** ***Δpxa1*** **-9LHP,** ***Δpxa2*** **-9LHP and** ***Δpxa1&2*** **-9LHP combined sample.**

The proteomic profiling of four functional strains was carried out by On-line 2D LC-MS/MS system (Additional file 1: Figure AF1). The Spectrum Mill system was used for peptides identification. Figure 2 and Additional file 1: Figure AF4 showed the representative peptide fragmentation spectrum of glucose-6-phosphate isomerase: (R)AVYHVALR(N). Basing on the analytical conditions, more than 200 proteins were detected while 31 showed different levels among the four functional strains, as shown in Table 2 with WT-9LHP as reference. The average of protein expression levels in WT-9LHP strain was taken as 1. The “average of B/A”, “average of C/A” and “average of D/A” refer to the average ratios of protein expression levels in Δ*pxa1*-9LHP, Δ*pxa2*-9LHP and Δ*pxa1&2*-9LHP strains over those in WT-9LHP strain.

**Table 2:** **Relative changes in protein expression between** ***S. cerevisiae*** **wild type and engineered strains**

Protein	Description	No. of peptides	Average of B/A	Average of C/A	Average of D/A
**Galactose metabolism**					
GAL1	Galactokinase	9	0.870 ± 0.340	0.587 ± 0.225	3.783 ± 0.215
GAL7	Galactose-1-phosphate uridylyltransferase	2	1.230 ± 0.005	1.387 ± 0.965	1.008 ± 0.021
**Glycolysis**					
HXK1	Hexokinase-1	2	0.723 ± 0.259	0.691 ± 0.104	3.489 ± 0.368
PGI1	Glucose-6-phosphate isomerase	3	1.032 ± 0.460	1.330 ± 0.180	9.891 ± 0.251
PFK2	Phosphofructokinase	3	1.236 ± 0.155	1.149 ± 0.100	1.426 ± 0.197
FBA1	Fructose-biophosphate aldolase	8	1.094 ± 0.155	0.871 ± 0.235	2.304 ± 0.942
TPI1	Triosephosphate isomerase	6	1.123 ± 0.380	0.849 ± 0.070	1.038 ± 0.357
TDH	Glyceraldehyde 3-phosphate dehydrogenase	15	1.117 ± 0.305	0.925 ± 0.220	1.664 ± 0.541
PGK1	Phosphoglycerate kinase	19	1.066 ± 0.225	0.798 ± 0.120	1.286 ± 0.076
GPM1	Phosphoglycerate mutase 1	14	1.047 ± 0.210	0.750 ± 0.145	1.500 ± 0.457
ENO	Enolase	19	1.185 ± 0.160	0.912 ± 0.155	2.176 ± 0.478
PYK1	Pyruvate kinase	6	1.186 ± 0.200	0.831 ± 0.195	1.831 ± 0.147
**TCA cycle**					
CIT1	Citrate synthase, mitochondrial	3	1.256 ± 0.820	1.982 ± 0.230	5.580 ± 0.248
ACO1	Aconitate hydratase, mitochondrial	3	0.961 ± 0.400	1.033 ± 0.075	1.737 ± 0.128
**ATP synthesis**					
ATP1	ATP synthase subunit alpha, mitochondrial	6	1.222 ± 0.260	1.005 ± 0.030	1.157 ± 0.160
ATP2	ATP synthase subunit beta, mitochondrial	8	1.176 ± 0.040	1.123 ± 0.085	3.216 ± 0.205
**Amino-acid metabolism**					
LEU1	3-isopropylmalate dehydratase	3	0.883 ± 0.075	1.023 ± 0.335	1.424 ± 0.200
LEU2	3-isopropylmalate dehydrogenase	17	3.477 ± 0.630	1.070 ± 0.335	2.570 ± 0.254
MET6	5-methyltetrahydropteroyltriglutamate--homocysteine methyltransferase	10	1.050 ± 0.125	0.881 ± 0.430	2.018 ± 0.121
PDC	Pyruvate decarboxylase isozyme	12	1.305 ± 0.355	1.118 ± 0.115	1.894 ± 0.218
**Protein biosynthesis**					
TIF	ATP-dependent RNA helicase eIF4A	3	1.408 ± 0.025	0.752 ± 0.295	1.655 ± 0.245
TEF1	Elongation factor 1-alpha	8	0.910 ± 0.375	0.758 ± 0.185	1.507 ± 0.110
RPL4	60s ribosomal protein L4	9	1.245 ± 0.255	0.778 ± 0.035	1.418 ± 0.068
RPL19	60s ribosomal protein L19	2	1.218 ± 0.285	1.114 ± 0.805	2.995 ± 0.197
**Heat shock proteins**					
HSP 12	12 kDa Heat shock protein	2	2.199 ± 0.640	0.882 ± 0.135	2.308 ± 0.219
HSP 26	Heat shock protein 26	3	2.281 ± 0.675	1.823 ± 0.360	2.453 ± 0.195
STI1	Heat shock protein STI1	2	1.363 ± 0.665	0.485 ± 0.035	3.450 ± 0.377
**Unknown**					
POR1	Mitochondrial outer membrane protein porin 1	4	1.033 ± 0.395	0.808 ± 0.445	2.785 ± 0.066
SAM2	S-adenosylmethionine synthetase 2	2	1.271 ± 0.300	0.624 ± 0.085	2.125 ± 0.151
YMR226C	Uncharacterized oxidoreductase YMR226C	2	1.856 ± 0.375	1.260 ± 0.110	3.051 ± 0.265
SOD1	Superoxide dismutase [Cu-Zn]	2	6.360 ± 0.420	3.942 ± 1.400	7.910 ± 0.330

Average of protein expression levels in WT-9LHP strain was taken as 1 and the deviation was calculated from three independent LC-MS/MS analysis results. The “Average of B/A” refers to the average ratio of protein expression level in Δ*pxa1*-9LHP strain over that in WT-9LHP strain. “Average of C/A”refers to the average ratio of protein expression level in Δ*pxa2*-9LHP strain over that in WT-9LHP strain. The “Average of D/A” refers to the average ratio of protein expression level in Δ*pxa1&2*-9LHP strain over that in WT-9LHP strain.

We classified the 31 protein of interest into eight categories according to their functions: GAL1 and GAL7 from galactose metabolism; HXK1, GI1, PFK2, FBA1, TPI1, TDH, PGK1, GPM1, ENO and PYK1 from glycolysis; CIT1 and ACO1 from TCA cycle; ATP1 and ATP2 from ATP synthesis; LEU1, LEU2, MET6 and PDC from amino-acid metabolism; TIF, TEF1, RPL4 and RPL19 from protein biosynthesis; HSP12, HSP26 from heat shock protein; POR1, SAM2, YMR226C and SOD1 involved in other bioprocess.

It is noteworthy that in the Δ*pxa1&2*-9LHP strain, all the proteins listed showed higher levels than strain WT-LHP to different extents. The levels of the listed proteins in strains Δ*pxa1*-9LHP and Δ*pxa2*-9LHP were mostly equivalent to strain WT-9LHP.

### Biotransformation

Functional strains and control strains were cultured in YNB-LEU selective media with galactose inducing the promoters of heterologous genes. As the produced 3(*Z*)-nonenal would be secreted outside and then vaporize into the headspace for being insoluble in water phase and volatile, 1 mL of the headspace of the cultures were extracted and injected into GC-FID for qualification and quantification.

Preliminary results have shown that, when linoleic acid was added to cultures of the growing cells, no detectable targeting volatile compounds was produced (Julsing et al. [2012]). Thus non-growing but metabolically-active resting cells with higher specific catalyzing activities were obtained in this study.

Functional strains WT-9LHP, Δ*pxa1*-9LHP, Δ*pxa2*-9LHP, Δ*pxa1&2*-9LHP and corresponding control strains WT-pESC, Δ*pxa1*-pESC, Δ*pxa2*-pESC, Δ*pxa1&2*-pESC were cultured, collected and prepared as resting cells for 3 days’ biotransformation. Gas samples of the headspaces of the cultures were determined with GC-FID system.

**Figure 3:**
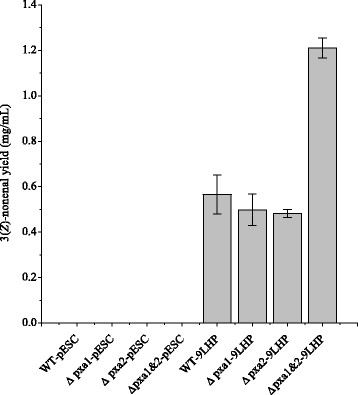
**3(** ***Z*** **)-nonenal production levels by functional strains.**

In the GC spectra, peaks at 8.82 min were identified as 3(*Z*)-nonenal (Additional file 1: Figure AF3). The characterizations of catalyzing activities of functional strains were repeated for 5 times, and the control strains were repeated for 3 times. Figure 3 showed the 3(*Z*)-nonenal production level. All the control strains produced non-detectable levels of 3(*Z*)-nonenal. WT-9LHP, Δ*pxa1*-9LHP and Δ*pxa2*-9LHP strains displayed similar producing capabilities, which were up to 0.57 ± 0.09 mg/L, 0.50 ± 0.07 mg/L and 0.48 ± 0.02 mg/L respectively. While Δ*pxa1&2*-9LHP produced twofold higher level of 3(*Z*)-nonenal, up to 1.21 ± 0.05 mg/L. The catalyzing efficiencies of the functional strains were also calculated. WT-9LHP, Δ*pxa1*-9LHP and Δ*pxa2*-9LHP have biotransformed 5.8%, 5.11% and 4.95% of linoleic acid into 3(*Z*)-nonenal respectively while Δ*pxa1&2*-9LHP performed the highest carbon recovery rate of up to 12.4% (Table 3).

**Table 3:** **Production of functional strains and carbon recovery rates**

Functional strains	WT-9LHP	Δ***pxa1***-9LHP	Δ***pxa2***-9LHP	Δ***pxa1&2***-9LHP
Yield (mg/L)	0.57 ± 0.09	0.50 ± 0.07	0.48 ± 0.02	1.21 ± 0.05
Carbon recovery (%)	5.80 ± 2.01	5.11 ± 1.63	4.95 ± 0.41	12.40 ± 0.05

## Discussion

Since the emergence of “metabolic engineering”, the potentials in producing unnatural specialty chemicals through genetic and metabolic modifications have been extensively explored, especially for the discovery of petroleum-replacing biofuels (Keasling [2012]). Among various reported biofuels, hydrocarbons, with high energy density and compatibility with current energy storage, transportation and utilization system, outstood as promising petroleum substitutes. While productions of short-chained (Atsumi et al. [2008]; Steen et al. [2008]; Santiago-Gomez et al. [2009]) and long-chained hydrocarbons (Schirmer et al. [2010]; Blazeck et al. [2013]) have been widely explored, biofuels and precursors in medium-chained range were seldom reported. In this study, we introduced hydroperoxide pathway to convert linoleic acid to 3(*Z*)-nonenal, one promising medium-chained hydrocarbon precursor.

The 2D LC-MS/MS approach was adopted to analyze the relative protein expression levels among four functional strains, WT-9LHP, Δ*pxa1*-9LHP, Δ*pxa2*-9LHP and Δ*pxa1&2*-9LHP as shown in Table 2. We classified the 31 protein of interest into eight categories according to their functions.

In order to induce the promoters on the recombinant plasmid, galactose was added to the culture medium as the sole carbon source. After absorption, galactose would be converted to glucose-1-phosphate to enter glycolysis through Leloir pathway. Two proteins, GAL1 and GAL7, which catalyze the two irreversible steps in Leloir pathway, showed comparable levels among WT-9LHP strain, Δ*pxa1*-9LHP strain and Δ*pxa2*-9LHP strain while up-regulated in Δ*pxa1&2*-9LHP strain.

Glycolysis is the metabolic pathway that converts glucose to pyruvate with the production of two molecules of ATP. Glycolysis pathway consists of ten enzymes: HXK1, PGI1, PFK2, FBA1, TPI1, TDH, PGK1, GPM1, ENO and PYK1. Our results showed that the levels of the ten enzymes in Δ*pxa1*-9LHP strain and Δ*pxa2*-9LHP strain were comparable to WT-9LHP strain. However, the levels of all the ten proteins were much higher (even 9.891 folds for PGI1) in Δ*pxa1&2*-9LHP strain which suggested that the up-regulated activity of glycolysis.

Pyruvate, the end product of glycolysis, can be used in aerobic respiration via TCA cycle. Pyruvate decarboxylated by pyruvate dehydrogenase catalyzing was converted into acetyl-CoA, the starting point of TCA cycle. Our results showed that, the mitochondrial enzymes CIT1 and ACO1 involved in TCA cycle and the enzymes ATP1 and ATP2 involved in ATP synthesis displayed equivalent or higher expression levels in Δ*pxa1*-9LHP strain, Δ*pxa2*-9LHP strain and Δ*pxa1&2*-9LHP strain comparing to WT-9LHP strain, suggesting the up-regulated activities.

The metabolic pathways mentioned above, galactose metabolism, glycolysis, TCA cycle an ATP synthesis, are steps in carbohydrate catabolism which breaks down carbohydrates and release energy in the form of ATP. It is noteworthy that the enzymes involved in these pathways were most notably up-regulated in the strain Δ*pxa1&2*-9LHP which suggested the most active metabolism and energy provision.

With galactose inducing the promoters, exogenous genes 9LOX and 9LHP carried by high copy number vector pESC were expressed. In the procedure of synthesis of unique proteins and peptides, amino acids metabolism was also important. LC-MS/MS results showed that four enzymes involved in the amino acid metabolism LEU1, LEU2, MET6 and PDC, along with four enzymes involved in protein biosynthesis TIF, TEF1, RPL4 and RPL19 were also significantly up-regulated in the strain Δ*pxa1&2*-9LHP. The up-regulations of these enzymes supported the exogenous genes expression well.

Proteins mentioned above were involved in energy-generation and protein synthesis. The significant higher levels in strain Δ*pxa1&2*-9LHP suggested possibly highest biotransformation efficiency. While for the strains WT-LHP, Δ*pxa1*-9LHP and Δ*pxa2*-9LHP, the expression level differences were slighter which suggested comparable biotransformation efficiencies. Our expectations would be tested in subsequent biotransformation studies performed on the functional strains and control strains.

Furthermore, three heat shock proteins related to stress response, HSP12, HSP26 and STI1 showed significantly different levels among the four functional strain. The introduction of exogenous genes and the folding of the proteins may well be stress to the yeast cells and the up-regulations of these proteins were to keep the balance of the intracellular metabolism. In addition, the levels of POR1, SAM2, YMR226C and SOD1 were also found different among the four functional strain which remained to be studied. The mechanism details of the above 31 proteins level differences however still need to be further investigated.

While resting cells showed good biotransformation activities, growing cells did not produce detectable amount of 3(*Z*)-nonenal. Possible explanation is that growing cells were more active in cell divisions rather than performing catalyzing reactions. Furthermore, with the presence of galactose in the culture, which is the preferred carbon source, growing cells would less likely to take linoleic acid from the medium.

In this study, single deletion strains displayed comparable biotransformation efficiency as the wild type strain, as shown in Figure 3. The significantly higher biotransformation efficiency of functional strain Δ*pxa1&2*-9LHP indicates that the combination of the two mutations would influence the flux of absorbed linoleic and further retain the absorbed linoleic acid in cytosol to be degraded through the introduced hydroperoxide pathway.

As in the biotransformation cultures, linoleic acid was the sole carbon source. Certain flux ratio would be degraded and generate energy to support the living activities of the cells apart from as substrate for 3(*Z*)-nonenal biotransformation,. Functional strains WT-9LHP, Δ*pxa1*-9LHP and Δ*pxa2*-9LHP performed equivalent biotransformation efficiencies, while Δ*pxa1&2*-9LHP strain showed two-fold higher biotransformation with an efficiency of up to 12.1%. This biotransformation results were consistent with our expectations from the proteomics analysis results.

In conclusion, we have demonstrated a yeast-based whole-cell biocatalyst capable of transforming polyunsaturated fatty acids into medium-chained aldehyde, the medium-chained biofuel precursor. The comparative proteomics analysis offered an approach to study the overall protein in the cells and potentials as catalyst. This study lay foundation in our future direction to synthesize medium-chained hydrocarbons through metabolic engineering approaches.

## Additional file

## Electronic supplementary material

Additional file 1: **Figure AF1.** Total intensity chromatogram results of peptides eluted by gradient concentrations of ammonium formate. **Figure AF2** Scheme of recombinant plasmid 9LHP. **Figure AF3** GC-FID spectra of biotransformation detection: retention time at 8.82 min was identified as 3(*Z*)-nonenal. Blue:3(*Z*)-nonenal standard; red: Δ*pxa1&2*-9LHP strain; green: Δ*pxa1&2*-pESC strain. **Figure AF4** LC-MS qualification results of representative peptide fragmentation spectrum of glucose-6-phosphate isomerase. **Table AF1** Heat map of proteomics results in Table. (DOC 182 KB)

Below are the links to the authors’ original submitted files for images.

Authors’ original file for figure 1

Authors’ original file for figure 2

Authors’ original file for figure 3

## References

[CR1] Atsumi S, Hanai T, Liao JC (2008). Non-fermentative pathways for synthesis of branched-chain higher alcohols as biofuels. Nature.

[CR2] Blazeck J, Liu LQ, Knight R, Alper HS (2013) Heterologous production of pentane in the oleaginous yeast *Yarrowia lipolytica*. J Biotechnol 165(3–4):184–194, 10.1016/j.jbiotec.2013.04.003 10.1016/j.jbiotec.2013.04.003 23602802

[CR3] Boundy B, Diegel SW, Wright L, Davis SC (2011). Biomass Energy Data Book.

[CR4] Chang MCY, Keasling JD (2006). Production of isoprenoid pharmaceuticals by engineered microbes. Nat Chem Biol.

[CR5] Feussner I, Wasternack C (2002). The lipoxygenase pathway. Annu Rev Plant Biol.

[CR6] Gietz RD, Schiestl RH (2007). Frozen competent yeast cells that can be transformed with high efficiency using the LiAc/SS carrier DNA/PEG method. Nat Protoc.

[CR7] Gueldener U, Heinisch J, Koehler GJ, Voss D, Hegemann JH (2002) A second set of *lox* P marker cassettes for Cre-mediated multiple gene knockouts in budding yeast. Nucleic Acids Res 30(6):e23, 10.1093/nar/30.6.e23 10.1093/nar/30.6.e23 PMC101367 11884642

[CR8] Hettema EH, Tabak HF (2000). Transport of fatty acids and metabolites across the peroxisomal membrane. Bba-Mol Cell Biol L.

[CR9] Hettema EH, van Roermund CWT, Distel B, vanden Berg M, Vilela C, RodriguesPousada C, Wanders RJA, Tabak HF (1996) The ABC transporter proteins Pat1 and Pat2 are required for import of long-chain fatty acids into peroxisomes of *Saccharomyces cerevisiae*. EMBO J 15(15):3813–3822 PMC452064 8670886

[CR10] Hiltunen JK, Mursula AM, Rottensteiner H, Wierenga RK, Kastaniotis AJ, Gurvitz A (2003) The biochemistry of peroxisomal beta-oxidation in the yeast *Saccharomyces cerevisiae*. Fems Microbiol Rev 27(1):35–64, 10.1016/S0168–6445(03)00017–2 10.1016/S0168-6445(03)00017-2 12697341

[CR11] Julsing MK, Kuhn D, Schmid A, Buhler B (2012) Resting cells of recombinant *E. coli* show high epoxidation yields on energy source and high sensitivity to product inhibition. Biotechnol Bioeng 109(5):1109–1119 10.1002/bit.24404 22170310

[CR12] Kaiser BK, Carleton M, Hickman JW, Miller C, Lawson D, Budde M, Warrener P, Paredes A, Mullapudi S, Navarro P, Cross F, Roberts JM (2013). Fatty aldehydes in cyanobacteria are a metabolically flexible precursor for a diversity of biofuel products. Plos One.

[CR13] Keasling JD (2012). Synthetic biology and the development of tools for metabolic engineering. Metab Eng.

[CR14] Lennen RM, Braden DJ, West RM, Dumesic JA, Pfleger BF (2010) A process for microbial hydrocarbon synthesis: overproduction of fatty acids in *Escherichia coli* and catalytic conversion to alkanes. Biotechnol Bioeng 106(2):193–202, 10.1002/Bit.22660 10.1002/bit.22660 PMC3833807 20073090

[CR15] Mita G, Gallo A, Greco V, Zasiura C, Casey R, Zacheo G, Santino A (2001) Molecular cloning and biochemical characterization of a lipoxygenase in almond (*Prunus dulcis*) seed. Eur J Biochem 268(5):1500–1507 10.1046/j.1432-1327.2001.02020.x 11231304

[CR16] Mita G, Quarta A, Fasano P, De Paolis A, Di Sansebastiano GP, Perrotta C, Iannacone R, Belfield E, Hughes R, Tsesmetzis N, Casey R, Santino A (2005). Molecular cloning and characterization of an almond 9-hydroperoxide lyase, a new CYP74 targeted to lipid bodies. J Exp Bot.

[CR17] Regalbuto JR (2009). Cellulosic biofuels-got gasoline?. Science.

[CR18] Rutherford BJ, Dahl RH, Price RE, Szmidt HL, Benke PI, Mukhopadhyay A, Keasling JD (2010) Functional genomic study of exogenous n-butanol stress in *Escherichia coli*. Appl Environ Microb 76(6):1935–1945, 10.1128/Aem.02323–09 10.1128/AEM.02323-09 PMC2838030 20118358

[CR19] Santiago-Gomez MP, Thanh HT, De Coninck J, Cachon R, Kermasha S, Belin JM, Gervais P, Husson F (2009) Modeling hexanal production in oxido-reducing conditions by the yeast *Yarrowia lipolytica*. Process Biochem 44(9):1013–1018, 10.1016/j.procbio.2009.04.028

[CR20] Santino A, Iannacone R, Hughes R, Casey R, Mita G (2005). Cloning and characterisation of an almond 9-lipoxygenase expressed early during seed development. Plant Sci.

[CR21] Schirmer A, Rude MA, Li XZ, Popova E, del Cardayre SB (2010). Microbial biosynthesis of alkanes. Science.

[CR22] Steen E, Chan R, Prasad N, Myers S, Petzold CJ, Redding A, Ouellet M, Keasling J (2008) Metabolic engineering of *Saccharomyces cerevisiae* for the production of n-butanol. Microb Cell Fact 7(1):36, 10.1186/1475–2859–7-36 10.1186/1475-2859-7-36 PMC2621116 19055772

[CR23] Steen EJ, Yisheng K, Bokinsky G, Zhihao H, Schirmer A, McClure A, del Cardayre SB, Keasling JD (2010). Microbial production of fatty-acid-derived fuels and chemicals from plant biomass. Nature.

[CR24] Tijet N, Schneider C, Muller BL, Brash AR (2001). Biogenesis of volatile aldehydes from fatty acid hydroperoxides: Molecular cloning of a hydroperoxide lyase (CYP74C) with specificity for both the 9-and 13-hydroperoxides of linoleic and linolenic acids. Arch Biochem Biophys.

[CR25] Wiese S (2007). Protein labeling by iTRAQ: A new tool for quantitative mass spectrometry in proteome research (vol 7, pg 340, 2007). Proteomics.

