# Supplementary material for: Proteomics analysis of metabolically engineered yeast cells and medium-chained hydrocarbon biofuel precursors synthesis
Source: AMB Express. 2014 Aug 21;4:61. doi: 10.1186/s13568-014-0061-8 (PMC4884031; doi:10.1186/s13568-014-0061-8)
Supplement: Supplementary file 1 — Additional file 1: Figure AF1. Total intensity chromatogram results of peptides eluted by gradient concentrations of ammonium formate. Figure AF2 Scheme of recombinant plasmid 9LHP. Figure AF3 GC-FID spectra of biotransformation detection: retention time at 8.82 min was identified as 3(Z)-nonenal. Blue:3(Z)-nonenal standard; red: Δpxa1&2-9LHP strain; green: Δpxa1&2-pESC strain. Figure AF4 LC-MS qualification results of representative peptide fragmentation spectrum of glucose-6-phosphate isomerase. Table AF1 Heat map of proteomics results in Table. (DOC 182 KB) [file 13568_2014_61_MOESM1_ESM.doc]

**Title: Proteomics Analysis of Metabolically Engineered Yeast Cells and Medium-chained Hydrocarbon Biofuel Precursors Synthesis**

**Journal: AMB Express**

**Authors:** Xiang Li and Wei Ning Chen

School of Chemical and Biomedical Engineering, Nanyang Technological University, Singapore, 637459

***Corresponding author:**

Prof. Dr. Wei Ning Chen

Professor of Biomolecular Engineering, School of Chemical and Biomedical Engineering

Director, 2nd Major in Food Science & Technology Programme

Co-ordinator, Graduate Education Unit, Nanyang Environment and Water Research Institute

Nanyang Technological University, 62 Nanyang Drive, N1.2-B1-07, Singapore 637459

DID: 6316-2870

Email: [WNChen@ntu.edu.sg](mailto:WNChen@ntu.edu.sg)

Web: [www.ntu.edu.sg/home/WNChen/William_Home.htm](http://www.ntu.edu.sg/home/WNChen/William_Home.htm)

**
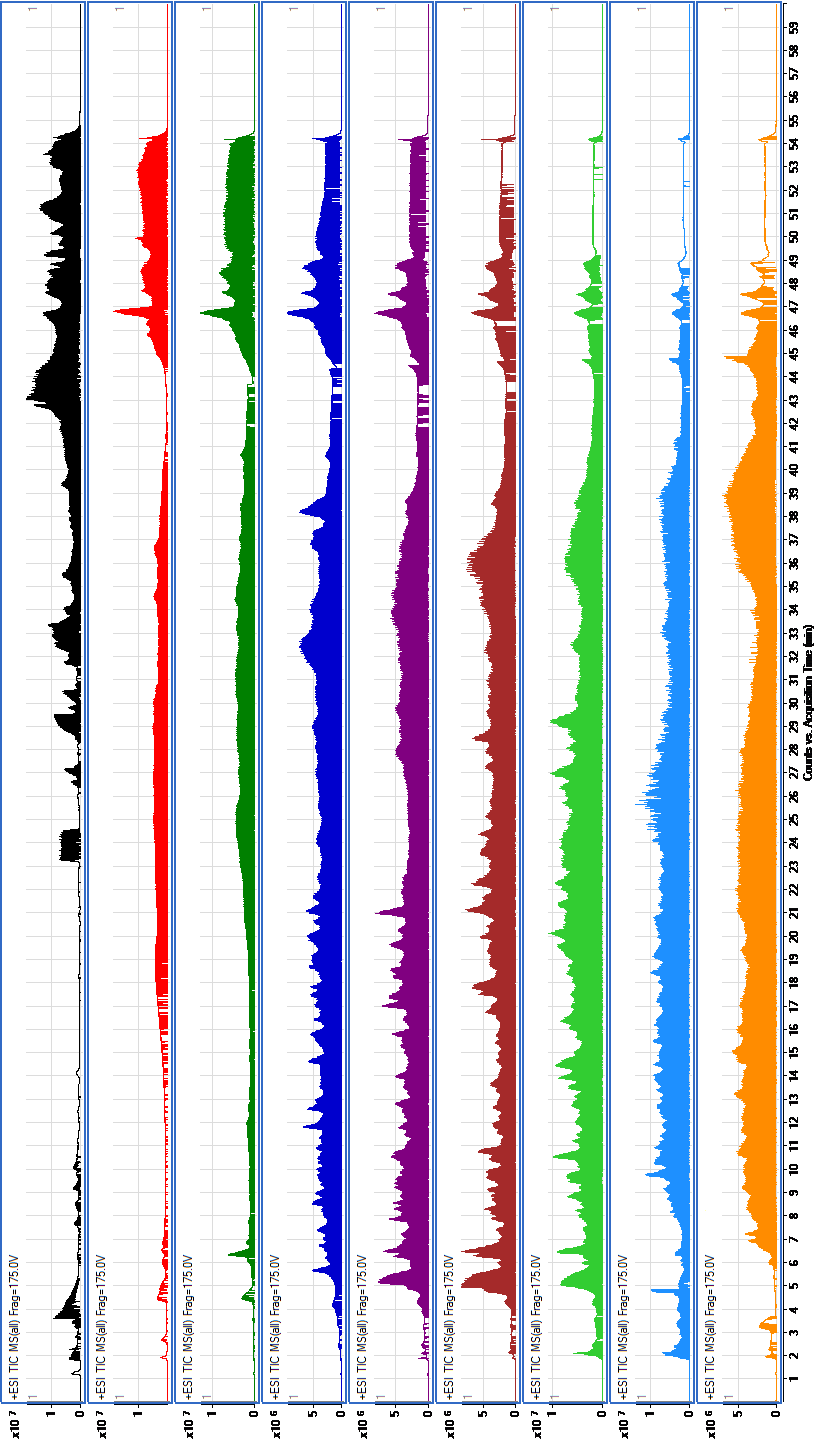
**

**Fig. AF1** Total intensity chromatogram results of peptides eluted by gradient concentrations of ammonium

formate
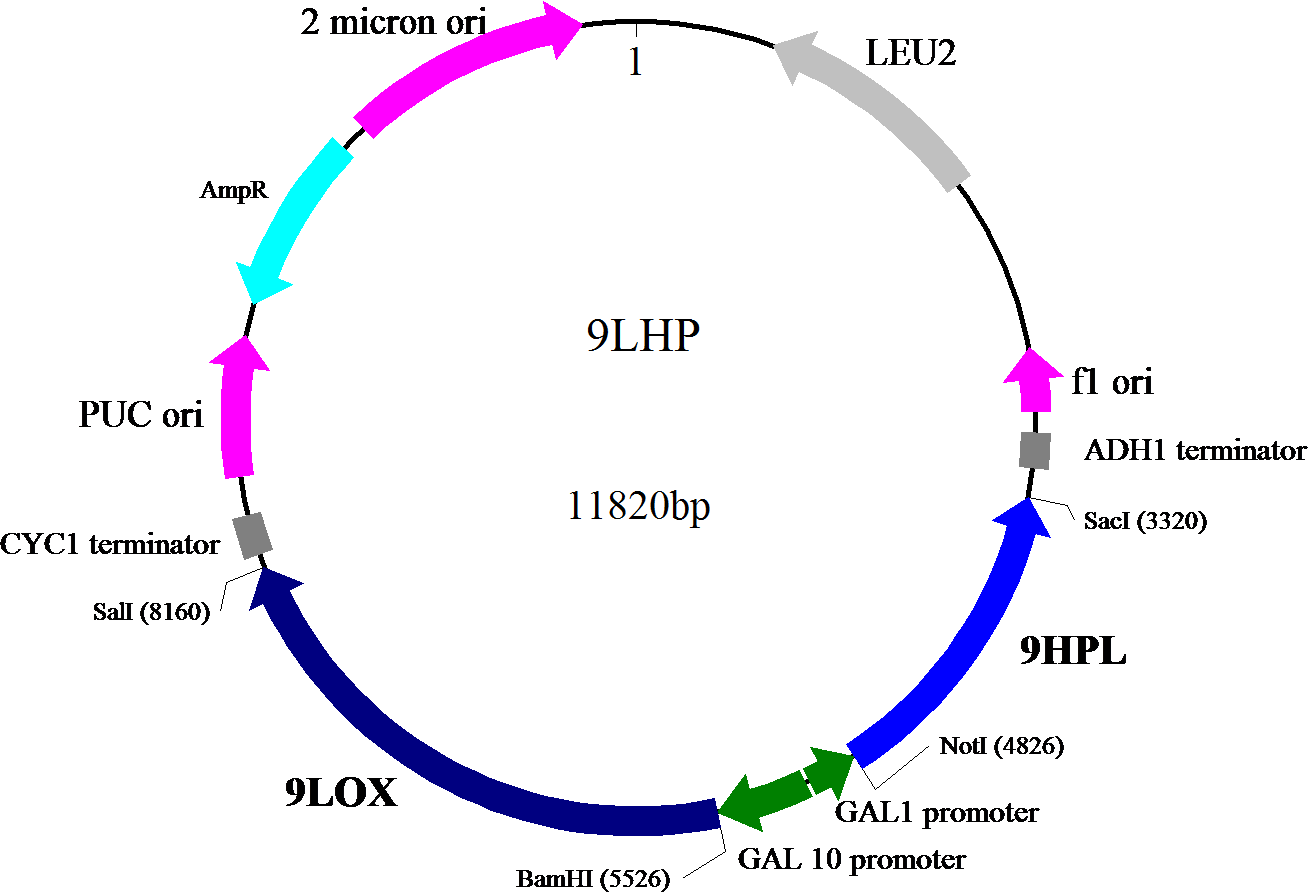


**Fig. AF2** Scheme of recombinant plasmid 9LHP


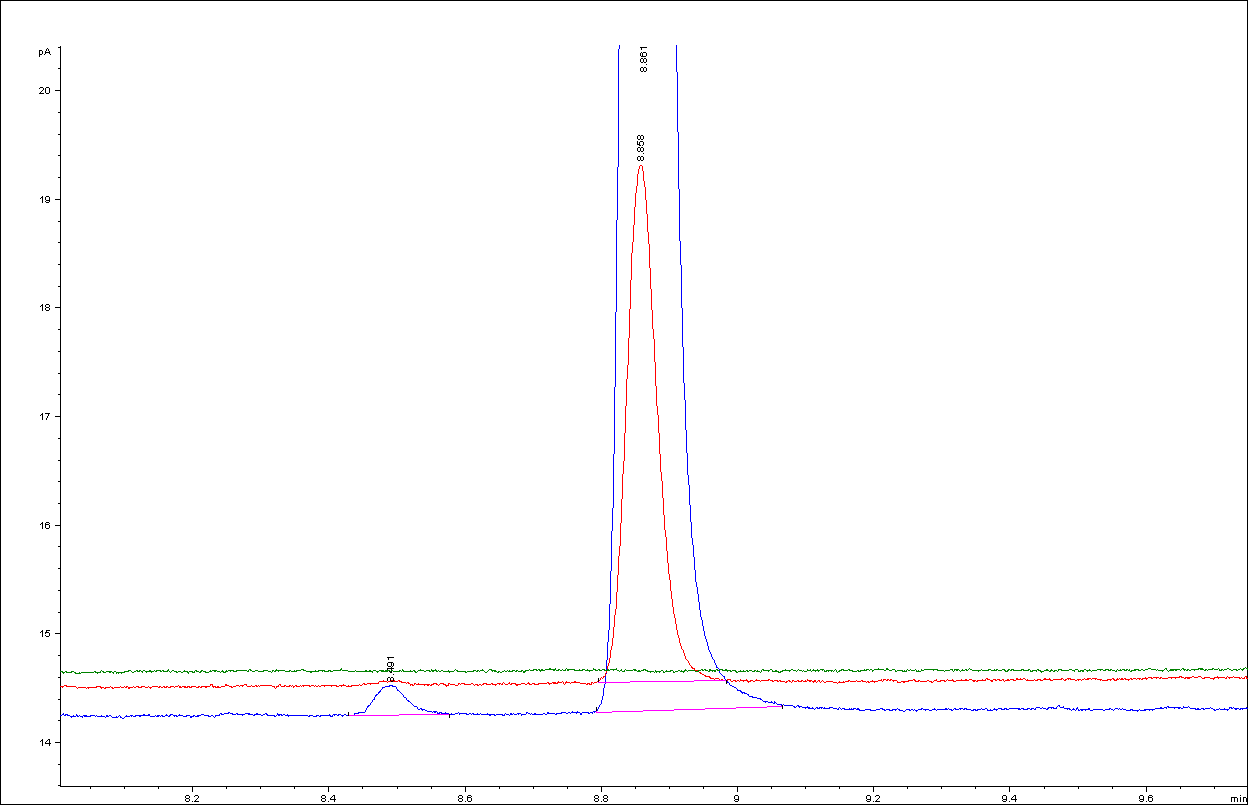


**Fig. AF3** GC-FID spectra of biotransformation detection: retention time at 8.82 min was identified as 3(*Z*)-nonenal. Blue:3(*Z*)-nonenal standard; red: Δ*pxa1&2*-9LHP strain; green: Δ*pxa1&2*-pESC strain.


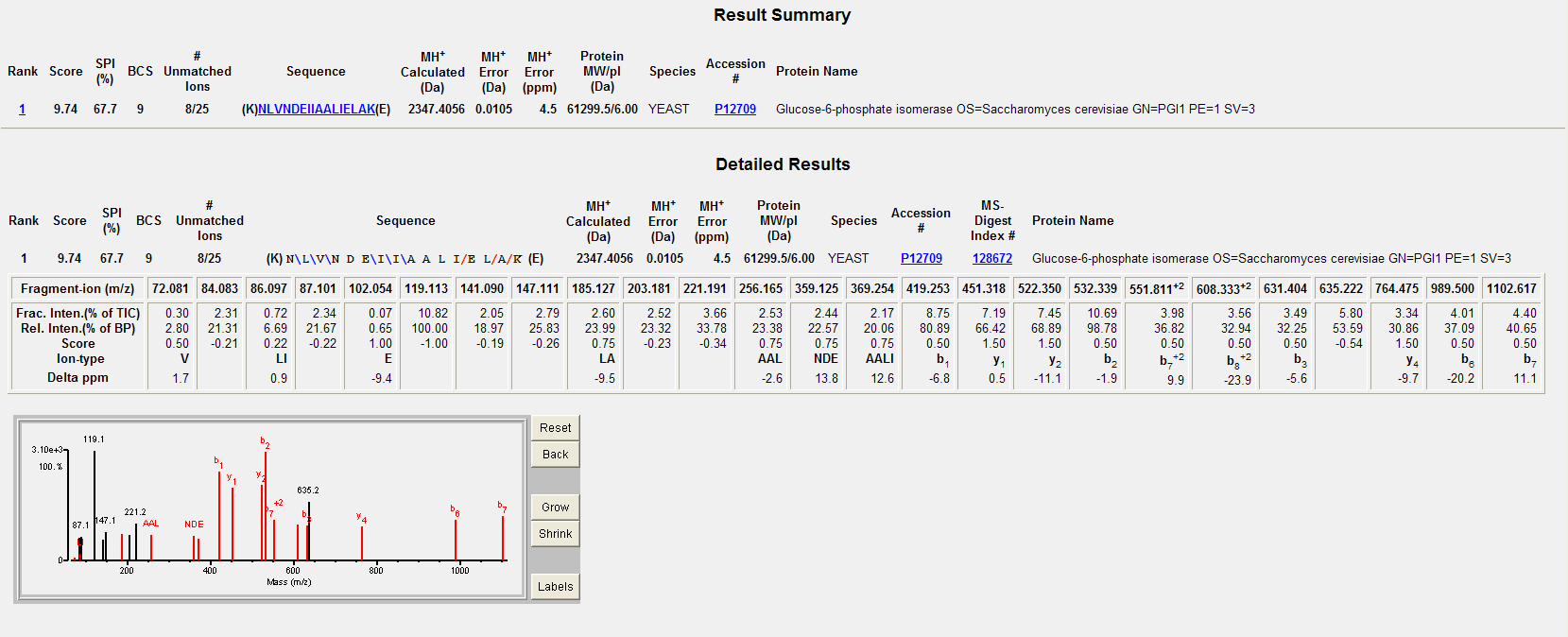


**Fig. AF4** LC-MS qualification results of representative peptide fragmentation spectrum of glucose-6-phosphate isomerase

**Table AF1** Heat map of proteomics results in Table 2

| Protein | Description | No. of peptides | Average of B/A | Average of C/A | Average of D/A |
| --- | --- | --- | --- | --- | --- |
| **Galactose metabolism** | | | | | |
| GAL1 | Galactokinase | 9 | 0.870 | 0.587 | 3.783 |
| GAL7 | Galactose-1-phosphate uridylyltransferase | 2 | 1.230 | 1.387 | 1.008 |
| **Glycolysis** | | | | | |
| HXK1 | Hexokinase-1 | 2 | 0.723 | 0.691 | 3.489 |
| PGI1 | Glucose-6-phosphate isomerase | 3 | 1.032 | 1.330 | 9.891 |
| PFK2 | Phosphofructokinase | 3 | 1.236 | 1.149 | 1.426 |
| FBA1 | Fructose-biophosphate aldolase | 8 | 1.094 | 0.871 | 2.304 |
| TPI1 | Triosephosphate isomerase | 6 | 1.123 | 0.849 | 1.038 |
| TDH | Glyceraldehyde 3-phosphate dehydrogenase | 15 | 1.117 | 0.925 | 1.664 |
| PGK1 | Phosphoglycerate kinase | 19 | 1.066 | 0.798 | 1.286 |
| GPM1 | Phosphoglycerate mutase 1 | 14 | 1.047 | 0.750 | 1.500 |
| ENO | Enolase | 19 | 1.185 | 0.912 | 2.176 |
| PYK1 | Pyruvate kinase | 6 | 1.186 | 0.831 | 1.831 |
| **TCA cycle** | | | | | |
| CIT1 | Citrate synthase, mitochondrial | 3 | 1.256 | 1.982 | 5.580 |
| ACO1 | Aconitate hydratase, mitochondrial | 3 | 0.961 | 1.033 | 1.737 |
| **ATP synthesis** | | | | | |
| ATP1 | ATP synthase subunit alpha, mitochondrial | 6 | 1.222 | 1.005 | 1.157 |
| ATP2 | ATP synthase subunit beta, mitochondrial | 8 | 1.176 | 1.123 | 3.216 |
| **Amino-acid metabolism** | | | | | |
| LEU1 | 3-isopropylmalate dehydratase | 3 | 0.883 | 1.023 | 1.424 |
| LEU2 | 3-isopropylmalate dehydrogenase | 17 | 3.477 | 1.070 | 2.570 |
| MET6 | 5-methyltetrahydropteroyltriglutamate--homocysteine methyltransferase | 10 | 1.050 | 0.881 | 2.018 |
| PDC | Pyruvate decarboxylase isozyme | 12 | 1.300 | 1.118 | 1.894 |
| **Protein biosynthesis** | | | | | |
| TIF | ATP-dependent RNA helicase eIF4A | 3 | 1.408 | 0.752 | 1.655 |
| TEF1 | Elongation factor 1-alpha | 8 | 0.910 | 0.758 | 1.507 |
| RPL4 | 60s ribosomal protein L4 | 9 | 1.245 | 0.778 | 1.418 |
| RPL19 | 60s ribosomal protein L19 | 2 | 1.218 | 1.114 | 2.995 |
| **Heat shock proteins** | | | | | |
| HSP 12 | 12 kDa Heat shock protein | 2 | 2.199 | 0.882 | 2.308 |
| HSP 26 | Heat shock protein 26 | 3 | 2.281 | 1.823 | 2.453 |
| STI1 | Heat shock protein STI1 | 2 | 1.363 | 0.485 | 3.450 |
| **Unknown** | | | | | |
| POR1 | Mitochondrial outer membrane protein porin 1 | 4 | 1.033 | 0.808 | 2.785 |
| SAM2 | S-adenosylmethionine synthetase 2 | 2 | 1.271 | 0.624 | 2.125 |
| YMR226C | Uncharacterized oxidoreductase YMR226C | 2 | 1.856 | 1.260 | 3.051 |
| SOD1 | Superoxide dismutase [Cu-Zn] | 2 | 6.360 | 3.942 | 7.910 |


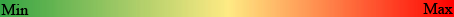
*Error bar can refer to Table 2. Color bar:
