# Supplementary figures and images for: Proteomics analysis of metabolically engineered yeast cells and medium-chained hydrocarbon biofuel precursors synthesis
Source: AMB Express. 2014 Aug 21;4:61. doi: 10.1186/s13568-014-0061-8 (PMC4884031; doi:10.1186/s13568-014-0061-8)

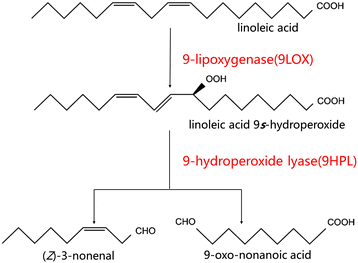

Supplement: Supplementary file 2 — Authors’ original file for figure 1 [file 13568_2014_61_MOESM2_ESM.gif]

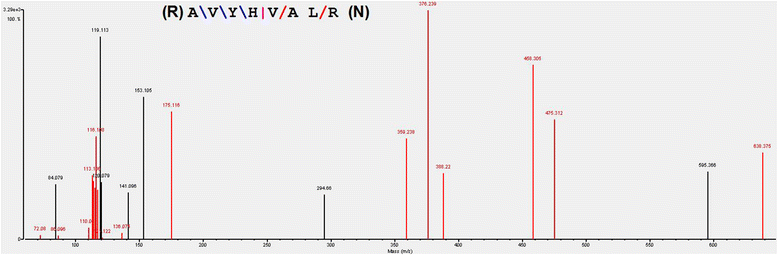

Supplement: Supplementary file 3 — Authors’ original file for figure 2 [file 13568_2014_61_MOESM3_ESM.gif]

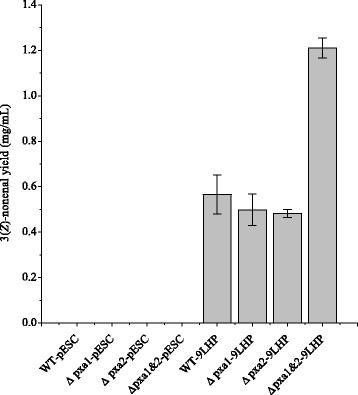

Supplement: Supplementary file 4 — Authors’ original file for figure 3 [file 13568_2014_61_MOESM4_ESM.gif]
